# B-Raf specific antibody responses in melanoma patients

**DOI:** 10.1186/1471-2407-4-62

**Published:** 2004-09-12

**Authors:** Joachim Fensterle, Jürgen C Becker, Tamara Potapenko, Veronika Heimbach, Claudia S Vetter, Eva B Bröcker, Ulf R Rapp

**Affiliations:** 1 Institut für Medizinische Strahlenkunde und Zellforschung (MSZ), University Clinics of Würzburg, Versbacher Str. 5, 97078 Würzburg, Germany; 2 Department of Dermatology, University Clinics of Würzburg, 97078 Würzburg, Germany

## Abstract

**Background:**

Mutations of the BRAF gene are the most common genetic alteration in melanoma. Moreover, BRAF mutations are already present in benign nevi. Being overexpressed and mutated, B-Raf is a potential target for the immune system and as this mutation seems to be an early event, a humoral immune response against this antigen might serve as a diagnostic tool for detection of high risk patients.

**Methods:**

372 sera of 148 stage IV melanoma patients and 119 sera of non-melanoma patients were screened for B-Raf, B-Raf V599E and C-Raf specific antibodies by an ELISA assay. Sera were screened for specific total Ig and for IgG. Serum titers were compared with a two tailed Mann-Whitney U test. Sera with titers of 1:300 or higher were termed positive and groups were compared with a two tailed Fisher's exact test.

**Results:**

B-Raf specific antibodies recognizing both B-Raf and B-Raf V599E were detected in 8.9% of the sera of melanoma patients and in 2,5% of the control group. Raf specific IgG was detected in some patients at very low levels. B-Raf specific antibody responses did not correlate with clinical parameters but in some cases, B-Raf antibodies emerged during disease progression.

**Conclusion:**

These findings imply that B-Raf is immunogenic in melanoma patients and that it might serve as a potential target for immunotherapy. However, B-Raf specific antibodies emerge at rather late stages of melanoma progression and are present only with a low frequency indicating that spontaneous B-Raf specific antibodies are not an early marker for melanoma, but rather may serve as a therapeutic target.

## Background

Cutaneous malignant melanoma is responsible for 1% of all malignant tumors with a rising incidence in the Caucasian population [1]. Initial diagnosis is based on asymmetry, border regularity, multiple colours, diameter as well as elevation of the pigmented lesion. However, it is sometimes difficult to differentiate between irregular dysplastic nevi and a melanoma without histological analysis. Hitherto risk groups for the development of melanoma are characterized by fair skin, multiple and/or dysplastic nevi and the history of sunburns in childhood [2]. Invasive melanomas have a rapid tendency to metastasize. In these stages of disease, therapy is very difficult and the 5-year survival rate of stage IV patients is below 20% [3].

On the molecular level, melanoma is associated with several genetic changes, including mutations or transcriptional variations in tumor suppressors like p53, CDKN2A/p16, CDKN1A/p21 or in oncogenes like N-Ras [4]. Recently, it has been discovered by Davies et al. that 66% of melanoma have a mutated BRAF gene which results in higher kinase activity due to a single amino acid exchange (B-Raf V599E) occurring in almost 90% of the mutations [5]. Most interestingly, this mutation is somatic [6,7] and some authors describe the presence of the mutation already in benign nevi [8], whereas others fail to reproduce the high frequencies in early stages and speculate of mutated B-Raf being relevant for progression rather than initiation of melanoma [9]. Nevertheless, the high incidence of the mutations in melanoma qualify the B-Raf protein as a potential target for tumor therapy and preliminary results of phase II clinical trials with Raf kinase inhibitors suggest protective activity [10].

Promising results with new approaches for melanoma therapy have been obtained with active immunizations against tumor associated antigens (TAA) like MAGE-3 [11], MART-1 [12-14] tyrosinase [15] or survivin [16]. Regarding the high incidence of B-Raf mutations and the increased expression level in tumors, B-Raf should be an attractive target for immunotherapy [17] and most recently, independent findings demonstrated mutation specific CD4^+ ^T-cell responses in melanoma patients [18]. Moreover, we have recently demonstrated CD8^+ ^T-cells in melanoma patients reactive against an HLA B27 restricted B-Raf V599E epitope encompassing the mutation identified using computer assisted algorithms [19]. However, assessing CD4^+ ^or CD8^+ ^T-cell responses is not suitable for screening larger numbers of patients to estimate responder frequencies.

To determine the frequency of B-Raf specific responses in melanoma patients and to evaluate whether B-Raf specific immune responses could serve as a melanoma marker we examined the Raf specific humoral response using an ELISA assay with purified recombinant B-Raf, B-Raf V599E or C-Raf protein.

## Methods

### Participants

Patient sera were obtained from frozen stocks and were collected over a period of 5 years. All patients gave informed consent to use their sera for scientific analysis. Control sera were obtained from patients of the dermatology department without signs of melanoma. Control patients were fully anonymized and no further information is available.

### Antigens for ELISA

Recombinant wild type B-Raf, B-Raf V599E and C-Raf proteins were expressed in Sf9 cells and purified as GST fusions (B-Raf V599E, C-Raf) or His-tagged proteins (B-Raf) as described [20]. Purity of the Raf kinase preparations was controlled by SDS-polyacrylamide gel electrophoresis and staining with Coomassie Blue.

### ELISA

372 sera of melanoma patients were analyzed for B-Raf V599E specific response, 271 sera were analyzed for B-Raf wt and C-Raf specific responses and 119 sera of non melanoma control patients of the dermatology department were used and analyzed for B-Raf V599E, B-Raf wt and C-Raf specific responses. For determination of serum levels of total Ig or IgG, an ELISA assay was developed. 1 μg/ml of purified B-Raf, B-Raf V599E or C-Raf protein in 100 μl coating-buffer or buffer alone as background control (carbonate buffer, pH 9,6) was coated on NUNC 96-Well MaxiSorp plates at 4°C overnight. Plates were washed twice with washing buffer (0,05% Tween (Sigma) in PBS) and blocked with 1% BSA (Sigma) in PBS. After washing twice, serial dilutions of human sera, starting at 1:100, in 100 μl conjugate buffer (1% BSA, 0,05% Tween in PBS) were incubated for 1.5 hours at 37°C. After four wash steps, alkaline phosphatase coupled goat anti human Ig (IgM + IgA + IgG, Dianova) or goat anti human IgG (Dianova) diluted 1:2000 in 100 μl conjugate buffer was added. After 1 h at 37°C and two washing steps, 50 μl of pNPP (Sigma) substrate in buffer was added. The reaction was incubated at room temperature and stopped after 20 min by 50 μl 1 M NaOH. Optical density was read at a wavelength of 405 nm (OD_405_) in a TECAN Spectra Thermo microplate reader.

For determination of specificity, a similar protocol was used, but plates were coated with different concentrations of antigen (0, 0.1, 1.0 and 2.0 μg/ml antigen in coating buffer). Each concentration was determined in duplicate. In addition, specificity was further confirmed using an unrelated antigen, prostate specific antigen (PSA, Sigma), at concentrations of 1 μg/ml for ELISA analysis. Two patients exhibited non-specific serum responses and were excluded from further analysis.

For competition ELISA performed with some positive sera, a similar protocol was engaged, using a fixed dilution of the sera and B-Raf or B-Raf V599E antigen for coating. Competition was performed by adding different concentrations of B-Raf or B-Raf V599E (0.0, 0.1, 1 or 2 μg/ml) to the human sera at the corresponding incubation step.

### Data analysis

For the analysis of the ELISA data, the titer of the sera was defined as the last serial dilution with an OD_405 _value exceeding the Cut Off 0.275, corresponding to the mean + 1.5 SD of the background values of positive sera at serum dilutions of 1:50. Raf specific titers were compared to the background values using a two tailed Mann-Whitney U test with a 95% confidence interval. The amount of positive sera within the two groups were defined as sera exceeding a given titer; the final definition of positive sera were sera with titers of 1:300 or higher. Frequencies of positive sera were compared using two sided Fisher's exact test with 95% confidence interval.

## Results and discussion

**Figure 1:**
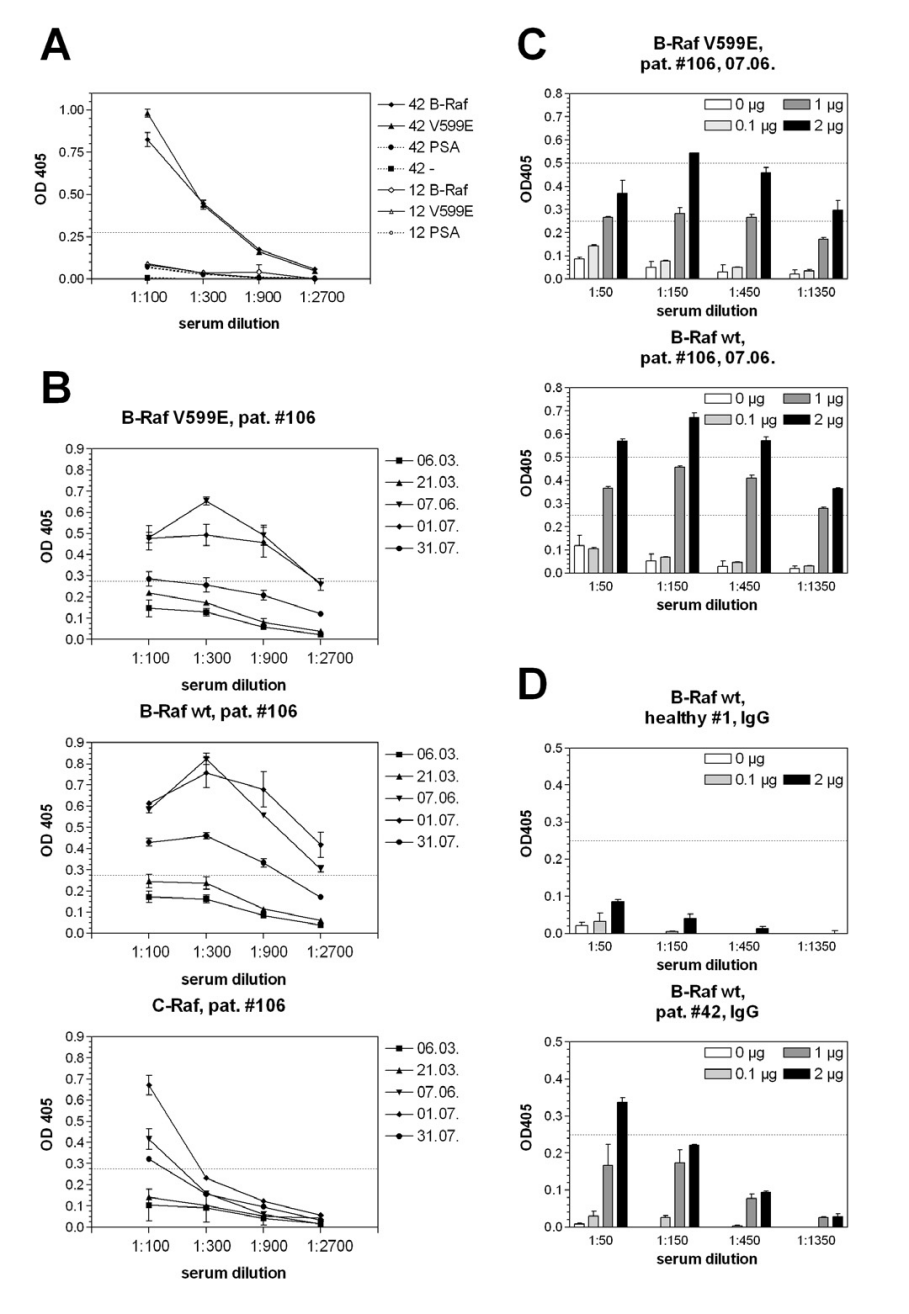
**Raf specific antibody response in different patients' sera. **Analysis of 2 representative positive (42) and negative (12) sera using a secondary antibody detecting human IgG, IgA and IgM is depicted in A. Sera no. 42 and 12 were tested for specificity against B-Raf, B-Raf V599E (V599E), an unrelated antigen (PSA) and without the addition of antigen (-). Serum 42 reacted against B-Raf and B-Raf V599E and not against an unrelated antigen. Sera 12 showed no measurable response. Sera withdrawn at different time points from patient no. 106 were tested at different dilutions in an ELISA and OD_405 _was determined (B). In the case of this patient, sera were negative at the first two time points and became strongly positive within three months. Specificity of the response was confirmed using different concentrations of antigen for coating (0, 0.1, 1 and 2 μg/ml) and different serum dilutions (C). All 371 patient and 119 control serum samples were analysed in a similar way. In D, serum was analyzed in a similar way then in C using an IgG specific secondary antibody. Serum #42 is weakly positive for B-Raf specific IgG antibodies, serum #1 is representative for most other sera of healthy donors and melanoma patients which are negative for B-Raf specific IgG antibodies.

**Figure 2:**
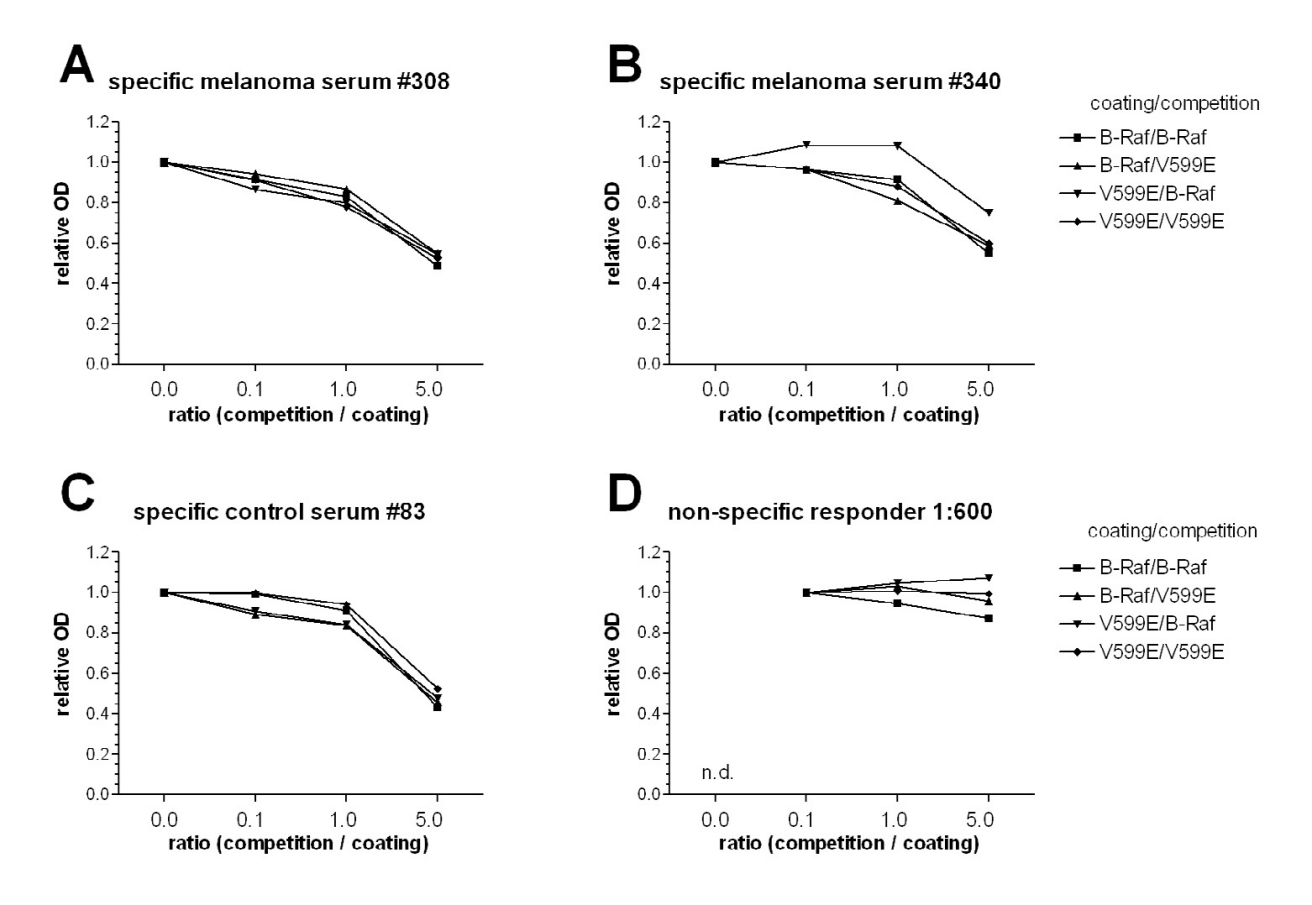
**Competition ELISA to test for specificity for mutated B-Raf. **ELISA was performed using 2 positive sera from melanoma patients (A, B) and 1 positive sera from the control group (C) as well as a human serum with known background reactivity (D; serum from a non-melanoma patient of the dermatology department with reactivity against BSA used for blocking). Plates were coated with B-Raf (■, ▲) or B-Raf V599E (▼, ◆) and competition was performed by titrating in increasing concentrations (0.0, 0.1, 1.0 and 5.0 μg/ml) of B-Raf (■ ▼) or B-Raf V599E (▲, ◆). Data is presented using relative OD_405 _values that are adjusted relative to the first experimental OD values of the corresponding dataset. For the positive sera with specific Raf antibodies (A-C), competition reveals no differences between B-Raf and B-Raf V599E regardless of the combination suggesting that the antibodies can not discriminate between wt and mutated B-Raf. In contrast, the non-specific serum no. 79 showed, as expected, no sign of competition with whatever antigen combination. n.d. not determined.

372 sera of 148 melanoma patients and sera of 119 control patients were screened for Raf specific antibody responses by an ELISA assay using a secondary antibody directed against IgG, IgA and IgM. Serial dilutions of the sera were analysed for their OD_405 _and titers were attributed as described using a cut off OD_405 _of 0.275 (fig. 1A and 1B). Positive sera reacted against B-Raf or B-Raf V599E, but not against an unrelated antigen (fig. 1A). Specificity was further confirmed by coating the ELISA plate using different concentration of the antigen (fig. 1C) and WESTERN Blot analysis (additional figure 1 [see additional file 1]). In some positive sera analysed for IgG antibodies, very weak B-Raf specific titers could be detected (fig. 1D), but most sera were negative for specific IgG antibodies (data not shown). Some positive sera were additionally tested for specificity for the mutated epitope using a competition ELISA by coating B-Raf or B-Raf V599E and competing Raf specific human antibodies by titrating in unbound B-Raf or B-Raf V599E (fig. 2A,2B,2C). In all cases tested, competition was achieved in every combination at corresponding antigen concentrations, whereas a control serum with non-specific reactivity (fig. 2D) showed no competition as expected. Taken together these results demonstrate the presence of Raf specific antibodies. In most cases, titers against B-Raf and B-Raf V599E were consistently higher compared to titers for C-Raf (fig. 1B); only some weakly positive sera showed a response dominated by C-Raf antibodies and in a minority of positive sera the specific response was equilibrated. Even though antigen preparations were not checked for biological activity and correct refolding and therefore a direct comparison between C-Raf and B-Raf specific antibody levels based on these assays is difficult, the data suggest that the Raf specific antibody response in patients is mainly directed against B-Raf. The moderate cross reactivity against C-Raf but not against an unrelated antigen might be explained by the high conformational and sequence similarity between Raf family members. In contrast, the differences observed between the reactivity against B-Raf or B-Raf V599E are weak (fig. 1B) and suggest that B-Raf and B-Raf V599E are recognized to a similar extend. This notion is further strengthened by the results of the competition ELISA which failed to detect any differences in the pattern of competition for a given serum regardless of the antigen combination used (figure 2). Therefore we conclude that the antibodies can not discriminate between the naïve or the mutated form of B-Raf.

**Figure 3:**
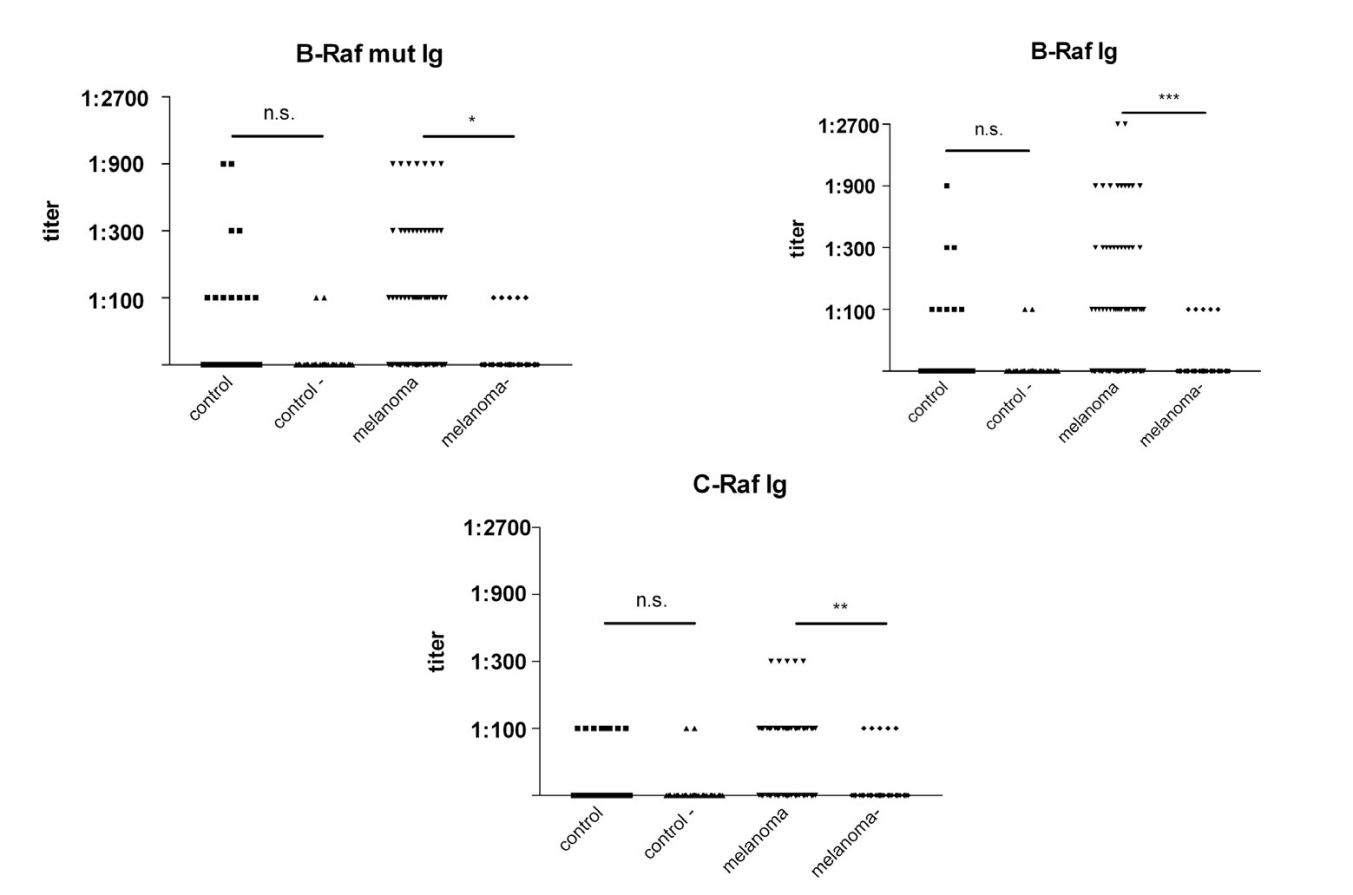
**Comparison of B-Raf V599E, B-Raf and C-Raf specific antibody responses between control patients or melanoma patients. **The comparison has been performed between 119 control sera with 371 sera of melanoma patients tested for B-Raf V599E and 272 sera of melanoma patients tested for B-Raf and C-Raf. Titers correspond to the last serial dilution of the patients sera exceeding a cut-off OD_405 _of 0.275. Compared to the background values in the absence of antigen (control -; melanoma -), the difference is not significant for control patients (P = 0.232; 0.471; 0.4054 for B-Raf V599E, B-Raf wt and C-Raf respectively, two tailed Mann-Whitney U test) and significant for melanoma patients (P = 0.012; 0.0009; 0.0022 for B-Raf V599E, B-Raf wt and C-Raf respectively, two tailed Mann-Whitney U test).

**Figure 4:**
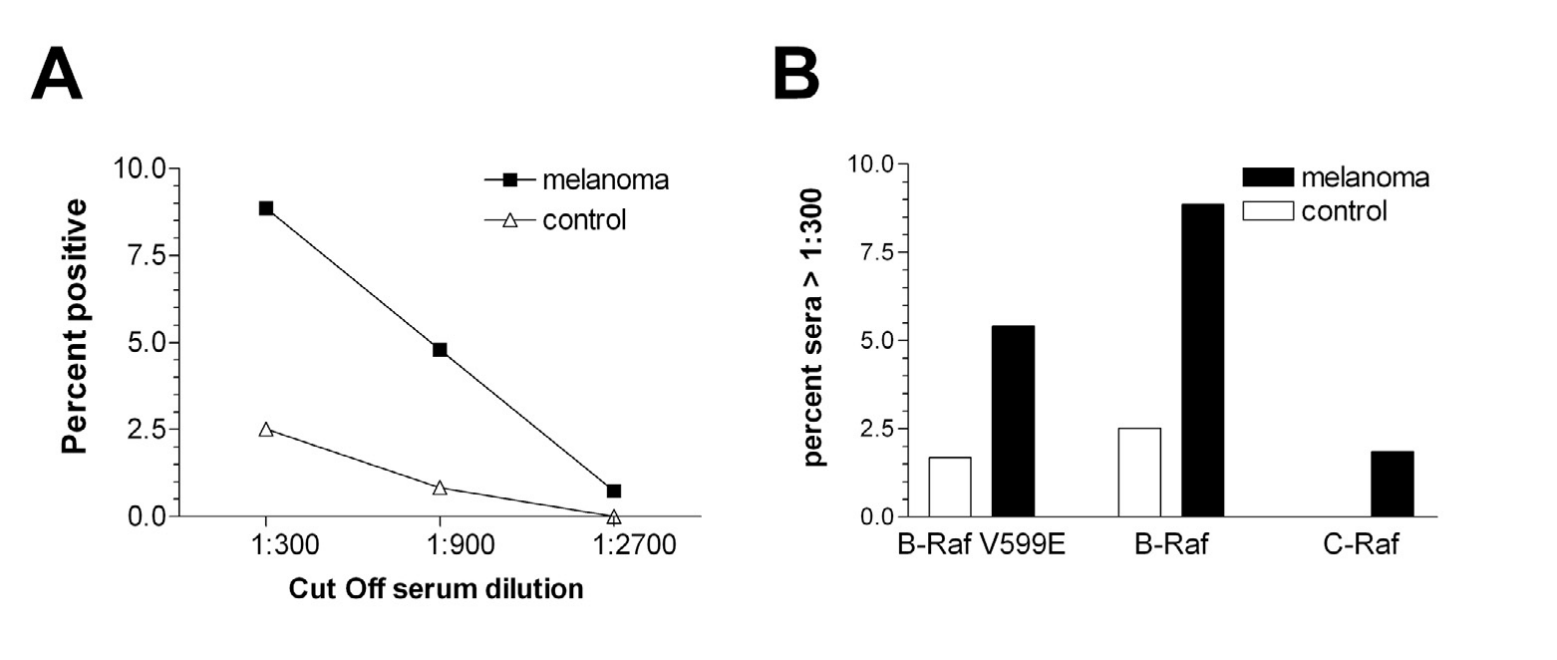
**Fraction of sera positive at different antibody levels. **In A, the percentage of B-Raf positive sera of control patients (△) or melanoma patients (■) is plotted as a function of the cut off titer, whereas sera are termed positive if the titer reaches or exacerbates the given cut off titer. The difference between control patients and melanoma patients was statistically significant for a cut off titer of 1:300 (P = 0.028; 0.073; 1.0, two sided Fisher's exact test for cut off titers equal to 1:300; 1:900 and 1:2700 respectively). In B, the percentage of sera with titers higher or equal than 1:300 is shown for B-Raf V599E, B-Raf and C-Raf specific responses. In all cases, the amount of antibody positive melanoma patients is higher compared to the control group. This trend was significant for the comparison of B-Raf positive sera (P = 0.028, Fisher's exact test) and not significant for the comparison of B-Raf V599E (P = 0.12, Fisher's exact test). However, C-Raf specific antibodies with titers of 1:300 were only detectable in 1.8% of the tested sera from melanoma patients and none of the control patients (P = 0.327).

Comparison between Raf specific antibody responses in sera from melanoma patients and control patients reveals apparent differences (figure 3). Raf specific responses are significantly different from background values for melanoma patients and all Raf variants tested (two tailed Mann-Whitney U test). In contrast, the difference observed within the control group is not significant (Mann-Whitney U test). However, reactivity against C-Raf is rather weak in all sera tested and only 5 of 271 tested sera from melanoma patients reach C-Raf specific titers of 1:300. A comparison between the percentage of patients with B-Raf specific antibody levels above a given titer reveals that sera derived from melanoma patients show consistently higher values compared to the control group (fig. 4A). In contrast, the frequency of C-Raf specific responses are only marginally higher in the melanoma population and not detectable at titers above 1:300. If positive response is defined as sera with titers of 1:300 or higher, 2.5% of the control group is positive for B-Raf and 1.68% for B-Raf V599E antibodies (fig. 4B). However, 5.41% (P = 0.12, Fisher's exact test) and 8.86% (P = 0.028, Fisher's exact test) of sera derived from melanoma patients were positive for B-Raf V599E and B-Raf respectively (fig. 4B). Using this cut off, no control patients could be identified with C-Raf specific antibodies, and only 1.85% (P = 0.329, Fisher's exact test) of the melanoma patients had detectable C-Raf specific antibodies (fig. 4B). These results suggest that melanoma is associated with higher rates of patients with detectable B-Raf specific antibody responses. The difference between the values obtained for B-Raf V599E and B-Raf specific antibodies, which is expressed in lower scores for B-Raf V599E, is most probably due to differences in the antigen preparation and is less likely due to different specificity as in most cases the scores against B-Raf V599E are in the same range as the scores against B-Raf (see figure 1B), whereas the C-Raf response consistently shows much lower titers in positive cases and a different shape of the curve (figure 1B). Taken together, these data strongly suggest that 8.9% of melanoma patients have a B-Raf specific antibody response and 2.5% of the control patients. At this stage, it is far too early to foresee the consequences of this finding for diagnostics or therapy of melanoma. All melanoma patients were in a very advanced, rapidly progressing stage with high tumor loads (stage IV) and consequently the presence of antibodies was not correlated with survival (data not shown). However, at least some patients had low but detectable levels of B-Raf specific IgG antibodies which is in line with the independent finding of B-Raf V599E specific T-cells in melanoma patients [18].

Another interesting point to note is the small, although not significant, number of control patients with detectable levels of Raf specific antibodies. At least two out of 119 control patients showed a very strong B-Raf specific response in the same range as we have observed for positive melanoma patients (figure 3). Due to the design of the study, no information is available for this patient group. Serum was sampled during diagnostic procedures, therefore other underlying diseases as a cause for Raf specific antibodies cannot be excluded, including autoimmune diseases or neoplastic diseases of different origin. Furthermore, as the control group has been sampled in the dermatology department, this group might contain patients with high numbers or irregular melanocytic nevi or even unrecognised melanoma.

For different extracellular tumor antigens, including gangliosides, in melanoma, positive associations between the induction of IgM and IgG antibodies and survival time have been reported [21] and the protective effect was attributed to antibody dependent cellular toxicity [22]. Therefore, the induction of Raf antibodies might be a favourable goal for the design of melanoma vaccines. However it is questionable whether humoral responses against an intracellular antigen like Raf will have an effect. In this respect it is interesting to ask why responses against intracellular antigens like Raf or survivin [23] can be observed at all. The most straightforward explanation, which is particularly likely for advanced stage IV melanoma patients, relies on the fact that the partial necrosis of big tumor masses allows the crosspresentation of intracellular proteins. The longitudinal analysis of our patients, e.g. the one depicted in figure 1, illustrates, that the occurrence of humoral immune responses was correlated rather to tumor burden than therapeutic measures, since the conversion occurred during the period when no therapy was applied.

As oncogenic mutation of B-Raf and even transformation by other oncogenic events is frequently accompanied by Raf overexpression, the induction of an antibody response is not necessarily due to the presence of the mutation. Even though a polyclonal IgM response is very unlikely to be specific for a point mutation, this notion would explain the lack of specificity for the mutational epitope. However, it is also possible that a cellular immune response prior to the induction of Raf specific antibodies has occurred. Such a T-cell response directed against Raf or other tumor antigens, would also result in the lysis of tumor cells and the liberation of intracellular antigens. It is important to mention that the lysis leading to the formation of antibodies against intracellular antigens can be caused by T-cells other than the already observed B-Raf V599E specific CD4^+ ^or CD8^+ ^T-cells. This question still remains to be clarified as up to now only advanced stage patients have been examined. It is obvious that at this stage the occurrence of antibodies is not correlated with prognosis and that it is very unlikely that the antibody response itself will have an influence on the disease. However, as it is rather unlikely that the frequency of a B-Raf specific T-cell response exceeds the frequency of antigen responses, our data can help to set the upper limit for the expected frequency of B-Raf or B-Raf V599E specific T-cells as 8.5% which allows the rational design of search strategies especially for Raf specific CD8^+ ^T-cells.

Even though the detection of B-Raf specific humoral and T-cell responses suggest that B-Raf/B-Raf V599E is immunogenic and that a response could be induced in at least a part of the patients, this notion does not allow conclusions on its suitability as a target for immunotherapy per se. In theory, an optimal tumor antigen is exclusively expressed in the tumor tissue and essential for tumor cell growth and survival to avoid the emergence of escape mutants or antigenic loss, whereas the immunological attack of the tumor cell is independent of the function of the antigen [17]. To date, many TAA used for the immunotherapy of melanoma including MAGE [24] or tyrosinase provide no obvious advantage to the tumor cell and therefore there is no pressure to retain the antigen. In contrast, mutated B-Raf has a high prevalence in melanoma and the consequences of its activation, including induction of proliferation and block of apoptosis are well known. However, it is still a matter of debate whether B-Raf activity mainly drives melanoma initiation or whether its function is also relevant for later stages. The first conclusion is supported by studies demonstrating that the mutations already occur in melanocytic nevi at frequencies comparable to late stage melanoma [8], are not correlated with clinical outcome [25] and finally the Raf inhibitor Bay 43-9006 seems to have only moderate efficiency in advanced melanoma as reported by Ahmed et al. during the ASCO meeting 2004 [26]. However, other studies correlated B-Raf mutations with progression rather then initiation [9] and we and others suggested a role for B-Raf as a negative prognostic factor in metastatic melanoma [25,27]. Despite these conflicting data on the clinical relevance of mutated B-Raf at late stages, the high prevalence would already justify to evaluate B-Raf/B-Raf V599E as a target for immunotherapy, as this prevalence is in the same range as for the TAA currently used without any evident advantage for tumor cell growth.

## Conclusions

Taken together we have demonstrated that B-Raf/B-Raf V599E specific antibodies are detectable in 8.9% of advanced stage melanoma patients and B-Raf V599E might therefore be a valuable target for immune therapy. Combined with the independently described B-Raf V599E specific CD4^+ ^and our earlier demonstration of B-Raf V599E specific CD8^+ ^T-cell responses this study makes the screening for novel B-Raf V599E MHC class I epitopes for vaccination approaches a promising task.

## Competing interests

None declared.

## Authors' contributions

JF and JCB designed the study and performed the data and statistical analysis. JF set up and supervised the ELISA and wrote the report. JCB set up and supervised the sample collection and was involved in writing the report. TP and VH were performing the ELISA assays, CSV was responsible for serum collection, transport and maintenance. EBB and URR were involved in providing the conceptual framework for this study. All authors approved the final version of the manuscript.

## Pre-publication history

The pre-publication history for this paper can be accessed here:



## Supplementary Material

Additional File 1 Supplementary figure with WESTERN Blot analysis of positive sera confirming specificity. Click here for file
