# Supplementary material for: B-Raf specific antibody responses in melanoma patients
Source: BMC Cancer. 2004 Sep 12;4:62. doi: 10.1186/1471-2407-4-62 (PMC518968; doi:10.1186/1471-2407-4-62)
Supplement: Additional File 1 — Supplementary figure with WESTERN Blot analysis of positive sera confirming specificity. [file 1471-2407-4-62-S1.pdf]

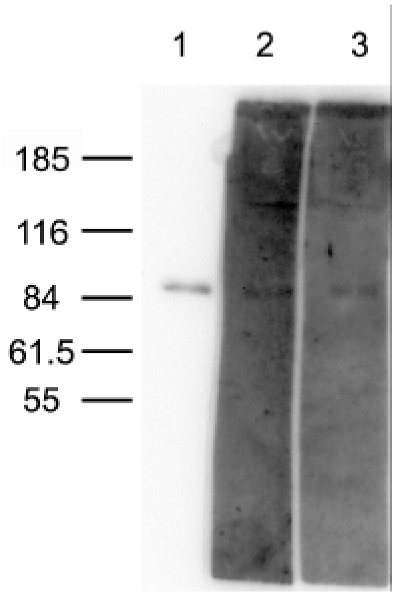

Supplementary figure 1 – WESTERN Blot analysis confirming specificity of ELISA assay

0.5 µg per lane purified B-Raf was separated by SDS PAGE and blotted onto a nitrocellulose membrane. The membrane was blocked using dry milk and incubated with B-Raf specific monoclonal antibody 12S (lane 1; dilution 1:5000) or with serum from patient number #106 taken at 07.06. (see fig. 1B and C) which was strongly positive in the ELISA assay (lane 2, 3; dilution 1:200). Membranes were washed and bound antibodies were detected using horseradish peroxidase coupled sheep anti mouse total Ig antibody (Amersham) diluted 1:1000 (lane 1) or horseradish peroxidase coupled goat anti human total Ig antibody (Dianova) diluted 1:1000 (lane 2) or 1:3000 (lane 3) and the ECL detection kit (Amersham).
